# 

**Published:** 1912-08-24

**Authors:** 


### Books Received.

Adlord and Co.: "L'Association Internationale d'Urologie."

Stanley Paul and Co.: "First Signs of Insanity," by R. Hollander.

Henry Frowde: "Stedman's Medical Dictionary," by Thomas Lathrop Stedman, A.M., M.D., 21s. net.

Association of Standardised Knowledge, Ltd.: "Axiom and Principles of the Science of Organisation," by Marshall Bruce-Williams, 7s. 6d.; "The Strategy of Nature," by Marshall Bruce-Williams, 2s. 6d.

### BUREAU OF INFORMATION.

### Rules for Correspondents.

1. Every letter, on whatever subject, **must be accompanied by the coupon** to be cut from the back cover (inside page) of The Hospital, current issue, and must contain the name and address of the correspondent with pseudonym for publication if desired.
2. Replies by post cannot be given, save under exceptional circumstances at the Editor's discretion.
3. Letters from Approved Homes in reply to special needs published in the Bureau should state terms and full particulars, and be sent prepaid under cover to the Editor of the Bureau with coupon and name enclosed for identification.
4. Proprietors of Homes which have not yet been entered on the List of Approved Homes, but have spare accommodation likely to suit special needs, are invited to write for an application form for registration. The fee for registration is 5s.
5. Special needs of every description relating to those who are sick or in difficulties are made known in these columns **free of charge.**
6. All communications to be addressed to The Editor of The Hospital, 28 Southampton Street, Strand, London, W.C., and marked "Bureau of Information."

### Training and Employment.

### Sanatorium Posts.

Vigilant.—You are right in surmising that there will be an opening for many officials in connection with the extension of sanatoriums throughout the country. We recommend you to study the advertisements in the professional papers, and if you have good testimonials and experience in this kind of work you might make formal application in advance to the Clerk of the Council at the County Hall in the district preferred.

### Free Training.

Middle Aged.—We cannot recommend you to apply for a short period of training with a view to becoming a village nurse, for you would find yourself handicapped by a two or three years' agreement at a low rate of pay. You would be better advised to enter a cottage hospital or institution for chronic cases and take the regular course.

### Hospital Almoners.

Kerry.—Apply to the Secretary, Hospital Almoners' Council, Denison House, 296 Vauxhall Bridge Road, London, S.W. From what you say you are well prepared for undertaking the additional training needed, the preliminaries to which are good education and some knowledge of social usages. Candidates must be under thirty-five. The training lasts from a year to eighteen months, during which time the candidate receives no salary, and must reside in or near London. A preliminary fee of £5 covers the out-of-pocket expenses. The training consists: first, in a course of instruction under the Charity Organisation Society in the routine work of an office and the business of dealing with applications for relief personally and by letter; secondly, in attendance at lectures on elementary hygiene and on social subjects; thirdly, in a course of practical experience under an almoner in an out-patients' department. When fully qualified the prospects of employment are good, the commencing salary being from £100 to £120 a year. It is hard work but intensely interesting.

### Sanitary Inspectors.

Gran.—Candidates for the technical examination of the Sanitary Inspectors' Board must be over twenty-one years of age, and must either have passed a preliminary examination or an equivalent, such as the Junior Oxford or Cambridge Examination. They must produce a certificate of instruction from an institution recognised by the Board (of which a list may be obtained from the Hon. Secretary, 1 Adelaide Buildings, London Bridge, E.C.), consisting of not less than thirty-two systematic lectures and demonstrations, including elementary chemistry and physics; building construction in its sanitary aspects; practical duties of a sanitary inspector. The fee for the technical examination is three guineas. The prospects are a little uncertain for female inspectors after qualification, but they are improving. Knowledge of nursing is certainly an asset in procuring appointments.

### Approved Homes.

Note.—*No Home is added to the List without direct medical and other testimony to its good management.*

### Change of Address.

Mrs. Roberts-Murray has removed from Rosebank to more commodious premises at 1 Esplanade Terrace, Bray, Co. Wicklow.

### Open-Air Home.

Greylands, Hatchend, Middlesex.—Patients received for change and rest or open-air cure. Nice garden; good balconies for out-door sleep, if desired. Quiet situation. Terms very moderate.

### Needs and Helpers.

### Home for Elderly Woman.

C. H. S.—Home required for elderly woman suffering from rheumatism and requiring good nursing. She can afford to pay 10s. a week but no more. Alas! good nursing in a private home is not to be had for these fees. If your friend could make it £1, or even 18s., we could find her accommodation. The homes for the aged do not as a rule take cases which require nursing unless there is hope of cure. Will one of our readers let us know if there is any opening for such a case?

### An Opening for a Home.

L. E. P.—We think it is quite possible that a private cripples' home where cases requiring continuous massage could be received for treatment by a trained nurse and masseuse might meet a public need. We shall be glad to hear from members of the profession and others as to their views on the need for such a home and the locality where it is likely to be of most use.

### Homes Wanted.

### For Lady with Partial Paralysis.

E. H. B., Yorkshire (118a).—We have no doubt we can find a suitable home for the lady you name who has had two apoplectic seizures and is now in a "hemiplegic" condition. We understand she has been a teacher all her life, and has only a small annuity. Let us know the utmost which can be paid. There are several homes of a charitable nature, but they seldom receive helpless cases, and your friend would be better with a nurse if even 25s. a week can be found.

### Letter-Box.

F. M. D.—We are sending on your letter *re* invalid child, and trust you will receive a favourable answer.

Clovelly.—If you will write a letter stating terms and accommodation we will forward it. See reply to E. H. B., Yorkshire.

J. H., Holly Park. We do not give addresses, but your letters are forwarded on without delay, and this comes to the same thing.

W. Peters.—We hope next week to let you have an answer to your query, which is not so simple as it appears.

**A leaflet containing particulars of the Bureau will be forwarded free on application.**

### The Editor's Letter-Box.

### "The Library."

*To the Editor of* The Hospital.

Sir,—Your correspondent, in his interesting article, "The Library," in the series of "Relaxations and Hobbies," is in error in saying (on p. 517 of The Hospital of August 17) that the minimum subscription to the London Library is £10. The subscription is £3 per annum.—Yours faithfully,

F. G. Haley.

National Liberal Club, Whitehall Place, S.W.
August 19.

### "The Seaford Mock-Marriage Case."

*To the Editor of* The Hospital.

### Appointments.

The Kent Insurance Committee has appointed Dr. H. O. West, of Queen Mary's Hospital, Carshalton, to be tuberculosis officer for the county. Dr. West was formerly house surgeon at King's College Hospital, London. A second tuberculosis officer is to be appointed at a similar salary (£500).

Mr. A. Ashley Cooper, L.R.C.P. and S. (Ireland), L.M., has been appointed resident medical officer to the Brixton Dispensary, in succession to Mr. V. Lindley Connolly, M.B., B.O.A., resigned. Mr. Ashley Cooper has recently been resident medical officer to the Chelsea Dispensary, which has been closed. His previous appointments include those of surgical registrar at Meath Hospital and County Dublin Infirmary and assistant medical officer at the Dublin Fever Hospital.

The recently created post of assistant surgeon for diseases of the throat, ear, and nose at Charing Cross Hospital has been filled by the appointment of Mr. Edward D. Davis. The previous appointments held by Mr. Davis include those of demonstrator of anatomy at Charing Cross Hospital Medical School, senior clinical assistant at the Hospital for Diseases of the Throat in Golden Square, and surgeon (nose, throat, and ear) at the Hostel of St. Luke in Fitzroy Square. He is a qualified dental surgeon.

### Gifts and Bequests.

The Middlesex Hospital Cancer Charity has received a further donation of £100 from Mr. E. S. Marcus towards the maintenance of the Cancer Investigation Department.

Sir Edwin Smith, of Australia, has given £1,000 to Walsall and District Hospital, which will probably be used to endow a bed in memory of the late Lady Smith.

Mr. Eimer Smith Judkins, of Hove, has left £100 to the Hove Dispensary, and £50 each to the Sussex County Hospital, Brighton, and the Alexandra Children's Hospital, Brighton.

Miss Catherine Clarke, of Rathmines, County Dublin, has bequeathed practically the whole of her estate to Roman Catholic charities in Ireland, including £1,000 each to the Hospice for the Dying, Harold's Cross, St. Patrick's Home, Kilmainham, under the care of the Little Sisters of the Poor, St. Vincent's Hospital, Stephen's Green, the Mater Misericordiæ Hospital, Eccles Street; £500 each to nearly thirty other institutions, and £300 each to twelve others. She also left eight premises, Nos. 127-134 (inclusive) Harold's Cross, County Dublin, to the Superioress of St. Clare's Orphanage, for the purposes of the said orphanage; a field at the rear of these houses to the Superioress of the Hospice for the Dying, Harold's Cross, directing that it shall not be built upon, but used as part of the grounds attached to the said hospital; and premises, No. 126 Harold's Cross, Rathmines, to the parish priest of Rathmines, to be used as a residence for the clergy acting as chaplains at the Hospice for the Dying and St. Clare's Orphanage. The residue of her estate she left for such charitable purposes in Ireland as the trustees of her will shall determine, directing that, should any of the legacies bequeathed by her will fail, the amount of such legacy or legacies shall be paid to the Roman Catholic Archbishop of Dublin.

Dr. William Francis O'Loughlin, who, as The Hospital noted at the time, was one of the medical men who went down in the *Titanic*, has left £2,412. He was in the regular service of the White Star Line.

